# Global phylogeography of the critically endangered hawksbill turtle (*Eretmochelys imbricata*)

**DOI:** 10.1590/1678-4685-GMB-2019-0264

**Published:** 2020-06-10

**Authors:** Larissa S. Arantes, Sarah M. Vargas, Fabrício R. Santos

**Affiliations:** 1 Universidade Federal de Minas Gerais (UFMG), Instituto de Ciências Biológicas, Departamento de Genética, Ecologia e Evolução, Belo Horizonte, MG, Brazil; 2 Universidade Federal do Espírito Santo (UFES), Instituto de Ciências Humanas e Naturais, Departamento de Ciências Biológicas, Vitória, ES, Brazil

**Keywords:** Sea turtles, population structure, mitochondrial DNA, phylogeny, gene flow

## Abstract

The hawksbill turtle is a broadly distributed, highly migratory and critically endangered sea turtle species. The paucity of studies restricts the comprehension of its behavior and life history. In this work, we performed a global phylogeographic analysis using a compilation of previously published mitochondrial haplotype data to understand the dynamics and diversity of hawksbill populations worldwide. Our results revealed a complex demographic pattern associated to hawksbill phylogeography since the Pliocene. Isolation by distance is not enough to explain distinct demographic units of hawksbill turtles, which are also influenced by other factors as oceanic currents, coral reef distribution and nesting timing. The foraging aggregations are typically mixed stocks of individuals originating from multiple nesting areas, but there is also a trend of foragers coming from nearby natal beaches. Phylogenetic analysis indicates two highly divergent major lineages split between Atlantic and Indo-Pacific rookeries, but there is also a more recent Atlantic Ocean colonization from the Indo-Pacific Ocean. Long-distance dispersal events are likely responsible for homogenization between distant populations within oceans. Our findings provided new insights about population connectivity, identified gaps that should be prioritized in future research and highlighted the need for international efforts aiming at hawksbill's conservation.

## Introduction

Species that exhibit worldwide distribution and highly migratory behavior are rarely studied in a global context, covering all population diversity and connectivity. As a result, only fragmented information is generated on the evolutionary history of species. Sea turtles are cosmopolitan animals that can disperse among different ocean basins during their lifetime (Bolten *et al.*, 1998; Monzón-Argüello *et al.*, 2011). Species dynamics change according to life stages, varying from strongly structured nesting populations or rookeries to highly diverse foraging aggregations or mixed stocks (Bowen *et al.*, 2005).

Phylogeographic studies use genetic and distribution data from populations to investigate processes related to their origin, distribution and dynamics (Beheregaray, 2008). Phylogeographic data has been especially important to provide current and historical information about sea turtle populations, allowing to characterize population structure and demographic history, to evaluate the remaining genetic diversity, to determine origins of individuals from foraging aggregations, to estimate gene flow and to comprehend oceanic migrations (Vilaça *et al.*, 2013; Proietti *et al.*, 2014; Shamblin *et al.*, 2014; Vargas *et al.*, 2016).

The majority of phylogeographic studies have used mitochondrial DNA (mtDNA) markers to study the genetic diversity of sea turtles (López-Barrera *et al.*, 2016), which allow recognizing different populations and tracking the relationships between rookeries. The control region (or D-loop) of the mtDNA is the baseline data for comparative analyses, but only local populations have been analyzed in detail, most commonly limited to one or few oceanic regions, undermining the access to the global population patterns. Moreover, diverse research groups have been performing genetic studies with sea turtles using independent methods and markers, resulting in incomparable data.

Efforts to compile control region haplotypes have been performed for some sea turtle species, such as loggerhead (*Caretta caretta*) and green turtles (*Chelonia mydas*), in a collaboration between research groups (Shamblin *et al.*, 2014) and The Archie Carr Center for Sea Turtle Research (http://accstr.ufl.edu/resources/mtdna-sequences). For *C. mydas*, a recent study investigated the global phylogeography using a compiled worldwide mtDNA dataset (Jensen *et al.*, 2019), and commitments to expand these models for other species are desired (Gaos *et al.*, 2016). In addition, international collaboration and the standardization of haplotype's nomenclature are critical to investigate sea turtle biology in a global context.

The hawksbill turtle (*Eretmochelys imbricata*) is a critically endangered species that occurs in tropical and subtropical waters around the world (Mortimer and Donnelly, 2008). The main threats to this species’ conservation are anthropogenic impacts, such as nesting area degradation, pollution, fisheries bycatch and exploitation through commercialization of the unique hawksbill shell, as well as meat and eggs (da Silva *et al.*, 2015). Threats have been intensified recently since hawksbill turtles preferred feeding areas are coral reefs, a greatly endangered marine ecosystem (Mortimer and Donnelly, 2008).

Phylogeographic studies of hawksbill turtles have been performed apart for populations in Brazil (Lara-Ruiz *et al.*, 2006; Vilaça *et al.*, 2013; Proietti *et al.*, 2014), Caribbean (Bass *et al.*, 1996; Troëng *et al.*, 2005; Bowen and Karl, 2007; Velez-Zuazo *et al.*, 2008; Blumenthal *et al.*, 2009; Richardson *et al.*, 2009; Browne *et al.*, 2010; LeRoux *et al.*, 2012; Carreras *et al.*, 2013; Wood *et al.*, 2013; Gorham *et al.*, 2014; Trujillo-Arias *et al.*, 2014; Cazabon-Mannette *et al.*, 2016; Hill *et al.*, 2018; Labastida-Estrada *et al.*, 2018), Eastern Atlantic (Monzón-Argüello *et al.*, 2010, 2011; Putman *et al.*, 2014), Indo-Pacific (Vargas *et al.*, 2016), Persian Gulf (Tabib *et al.*, 2011, 2014; Natoli *et al.*, 2017), Eastern Pacific (Gaos *et al.*, 2012, 2016, 2017, 2018; Zuniga-Marroquin and Monteros, 2017), Southeast Asia (Nishizawa *et al.*, 2016) and Japan (Nishizawa *et al.*, 2010). The first genetic studies used mtDNA control region data based on approximately 300 bp sequences (Bass *et al.*, 1999; Dias-Fernández *et al.*, 1999; Troëng *et al.*, 2005; Bowen and Karl, 2007; Nishizawa *et al.*, 2010). More recently, longer sequences began to be used, increasing the detection of genetic variation and improving the inference power of population structure and connectivity among distant populations (LeRoux *et al.*, 2012).

In a global perspective, there is a deep genetic divergence between hawksbill's lineages from Atlantic and Indo-Pacific basins. The divergence time between oceanic lineages has been previously dated to the Pliocene epoque, when the closing of the Isthmus of Panama occurred (Duchene *et al.*, 2012). The American and African continents are both considered important barriers to species migration, as the hawksbill turtle is adapted to tropical waters and rarely may reach such high latitudes. However, a potential gene flow has been reported from the Indo-Pacific to the Atlantic basin (Monzón-Argüello *et al.*, 2011; Vilaça *et al.*, 2013).

Within the oceanic basins, hawksbill populations also exhibit high levels of genetic diversity, but an evident population structure between rookeries was not observed (LeRoux *et al.*, 2012; Vargas *et al.*, 2016). Therefore, the phylogeographic pattern of the species is complex, likely involving weak isolation by distance and periodic long-distance colonization events.

The connectivity between populations is also influenced by oceanic currents, which likely drive the formation of foraging aggregations (Blumenthal *et al.*, 2009). The mixed stocks in these areas comprise a variety of individuals that come from distant rookeries under the influence of currents. Few cases of long-distance dispersals were registered, including transoceanic migrations (Monzón-Argüello *et al.*, 2010; Vilaça *et al.*, 2013; Putman *et al.*, 2014). Most of these conclusions were achieved by comparing mtDNA haplotypes between rookeries and mixed stocks that have been genetically described through a Bayesian method of mixed stock analysis (MSA), but the lack of standardized data makes these analyses extremely difficult.

Management units (MU) have been recognized as populations with a significant divergence of allele frequencies considering current population structure and management issues (Moritz, 1994). Sea turtle studies suggested the establishment of different MUs based on mtDNA variation found for each analyzed sample (Velez-Zuazo *et al.*, 2008; Vargas *et al.*, 2016; Gaos *et al.*, 2017). Wallace *et al.* (2010) developed the idea of regional management units (RMU) on a global scale, which considers nesting sites, genetic stocks and geographic distributions. For hawksbills, 13 RMUs were identified worldwide, of which seven were designed as putative RMUs due to the lack of genetic or distribution data. A subsequent study was based on these delineations to identify knowledge gaps and to evaluate the conservation status of sea turtles (Mazaris *et al.*, 2017).

Thus, comparative studies exploring all population diversity and linking separated studies are essential to comprehend the biology, behavior and life history of hawksbill turtles. In this work, we compiled a complete dataset of all previously published mtDNA data with a standardized nomenclature to analyze the global phylogeography in order to investigate the hawksbill turtle dynamics and diversity worldwide.

## Material and Methods

### Data Collection

We compiled all control region mtDNA haplotypes data available in the literature for hawksbill turtles. The sequences were obtained from the GenBank Database (www.ncbi.nlm.nih.gov, accessed 11 February 2019) and from the Atlantic Ocean hawksbill haplotype database (A. Abreu-Gobrois, personal communication). According to the purpose of this work, only haplotypes with associated population frequencies data were recovered, including females nesting in rookeries and juveniles in foraging aggregations. Populations with less than three individuals analyzed were grouped with the closest population (<300 km). We analyzed control region haplotypes spanning 739 bp, aiming to increase detection of variation and population structure. We standardized the nomenclature to abbreviation EiA plus sequential number, when the haplotype was first detected in Atlantic Ocean, and EiIP plus sequential number, when the haplotype was first recorded in Indo-Pacific Ocean. The alignment of sequences was performed using ClustalX algorithm of the MEGA 7 software (Kumar *et al.*, 2016).

### Phylogenetic analysis

The divergence time estimates between major mtDNA lineages described for hawksbill turtles was calculated using the software BEAST v2.4.3 (Bouckaert *et al.*, 2014). The choice of the best nucleotide substitution model was performed in jModelTest v. 2.1.7 (Posada, 2008) using a gamma distribution with four rate categories and Bayesian information criteria (BIC). The best model was TrN+G+I with gamma shape=0.71 and proportion invariable=0.75.

We assumed rate homogeneity among branches (strict molecular clock) under the Coalescent Bayesian Skyline Population tree model due to the intraspecific nature of the dataset (Drummond and Bouckaert, 2014). The times to the most recent common ancestors (TMRCAs) based on previous genetic studies (Duchene *et al.*, 2012; LeRoux *et al.*, 2012; Vargas *et al.*, 2016) were used as priors for tree calibration, assuming the monophyly of the group. The TRMCA prior used for the root age of the tree was 5.63 million years ago (mya) with a 95% confidence interval of 3.98–8.86 mya.

We conducted three independent runs for 200,000,000 generations, sampled every 1000 generations. Trace files were checked for chain convergence and sufficient effective sample sizes (ESS) in Tracer v. 1.6 (ESS>200 were considered acceptable) (Rambaut *et al.*, 2014). TreeAnnotator v2.4.2 was used to find the maximum clade credibility (MCC) tree within all trees generated.

### Genetic diversity and population structure analyses

We estimated the number of haplotypes, haplotype diversity, nucleotide diversity (per site), number of variable sites and average number of nucleotide differences using the program DnaSP v5 program (Librado and Rozas, 2009). The parsimony relationship among haplotypes was represented in a network using the median-joining algorithm (Bandelt *et al.*, 1999) in the software PopART 1.7 (Leigh and Bryant, 2015).

The level of differentiation between populations was estimated using the population pairwise FST values based on haplotype frequencies and exact tests of population differentiation in the software Arlequin v3.5 (Excoffier and Lischer, 2010). We also used the spatial analysis of molecular variance (SAMOVA) to find the most likely demographic groupings, whose approach is based on the maximization of the proportion of total genetic variance due to the differences between groups (FCT). The analysis was conducted for the complete dataset and separately for each oceanic basin in the program SAMOVA 2 (Dupanloup *et al.*, 2002). The largest mean FCT value is associated with the estimated number of simulated groups. This simulated annealing approach was performed testing the number of populations groups (K) from two to 15. Aldabra was not included in the statistical analyses due to the small number of individuals and distance from the other rookeries. To test for demographic expansion, we performed neutrality tests (Tajima's D and Fu's Fs) with the software Arlequin v3.5 (Excoffier and Lischer, 2010).

## Results

Haplotype frequencies were obtained from the literature for 18 rookeries and 23 foraging aggregations from Atlantic Ocean and for 22 rookeries and 17 foraging aggregations from Indo-Pacific Ocean. We analyzed a total of 1983 individuals from worldwide rookeries that exhibited 88 haplotypes and 1577 individuals from foraging aggregations that displayed 79 haplotypes. The global dataset including all mtDNA control region haplotypes previously recorded for hawksbill turtles is presented in Table 1. The variable positions for control region haplotypes, Genbank access numbers, corresponding haplotypes based on shorter sequences (384 bp) (Bass *et al.*, 1996; Dias-Fernández *et al.*, 1999) and ambiguous names for haplotypes are available in Table S1.

**Table 1:** Dataset of haplotypes based on mitochondrial control region (739 bp) of the hawksbill turtles and genetic diversity of rookeries from Atlantic and Indo-Pacific Ocean. R: number of rookeries; N: number of individuals; H: number of haplotypes; S: number of polymorphic sites; π: nucleotide diversity (per site); K: average number of nucleotide differences; FA: number of foraging aggregations.

Ocean Basin	Rookeries						Foraging aggregations		
	R	N	H	S	π	K	FA	N	H
Atlantic	18	992	27	23	0.009	6.684	23	904	45
Indo-Pacific	22	991	61	69	0.022	16.144	17	673	35
Total	40	1983	88	–	–	–	40	1577	79

Several ambiguities in the nomenclature of mtDNA haplotypes were found (Table S1). For example, haplotypes EiBR14 (Vilaça *et al.*, 2013), EiA67 (Proietti *et al.*, 2014) and EiIP33 (Vargas *et al.*, 2016) are equivalent to same 739 bp sequence haplotype. In this work they were treated as EiIP33, as it is related to the major Indo-Pacific lineage. The haplotypes EiBR7 (Vilaça *et al.*, 2013), EiA48 (Putman *et al.*, 2014) and EiIP16 (Vargas *et al.*, 2016) are also equivalent and here they were named EiIP16, since it is also related to the major Indo-Pacific lineage. Haplotypes Ei_15 (Nishizawa *et al.*, 2016) and EiIP17 (Vargas *et al.*, 2016) correspond to the same 739 bp haplotype that is called EiIP17 in this work. We also found a miscalling in haplotypes EiA23 and EiA41 deposited in the GenBank database by LeRoux *et al.* (2012) (confirmed by personal communication). The haplotype EiA23 (Genbank accession number JN998521) corresponds to EiA41 (GenBank acc. no. EF210793), and EiA41 (Genbank acc. no. JN998517) corresponds to EiA23 (GenBank acc. no. EF210791) according to Velez-Zuazo *et al.* (2008) and to the Atlantic Ocean hawksbill haplotype database (Table S1).

**Figure 1:**
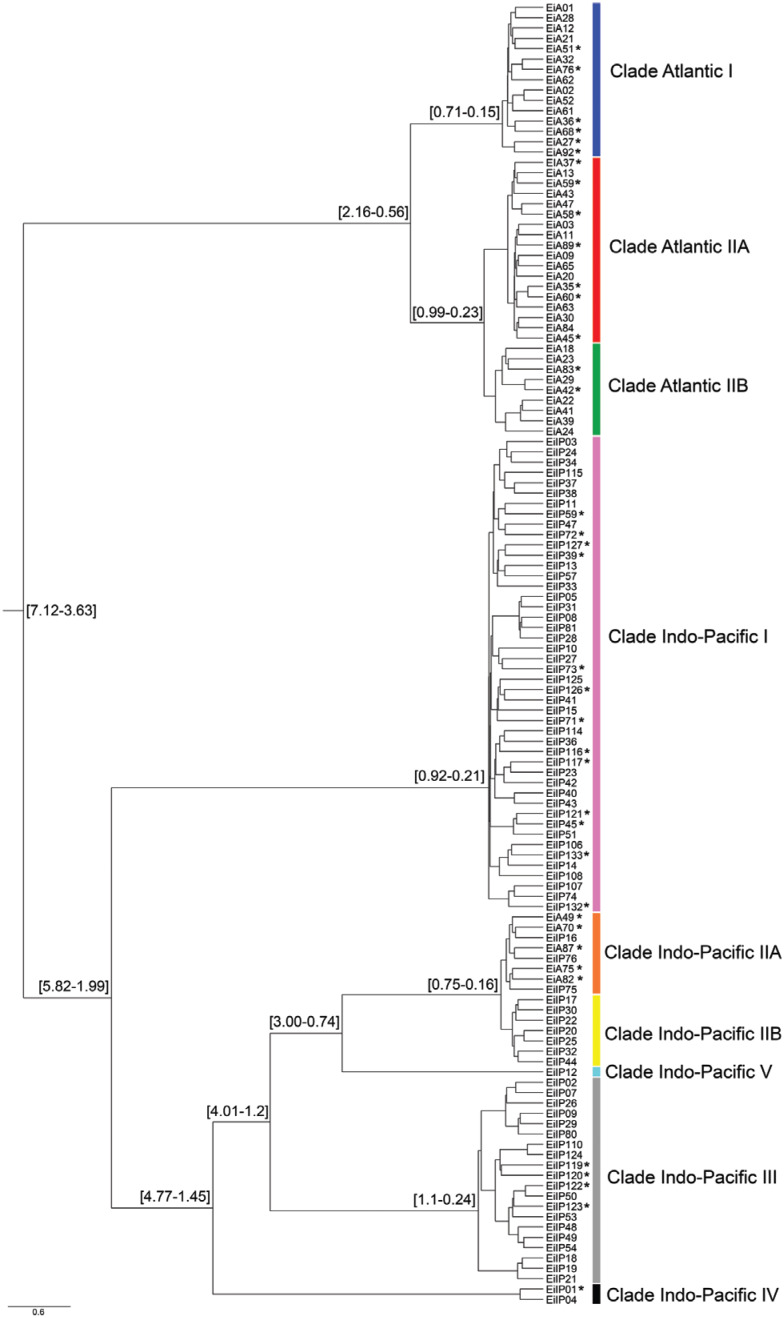
Bayesian tree based on unique mitochondrial control region haplotypes (739 bp) of hawksbill turtles reported in literature from rookeries and foraging aggregations worldwide. The 95% highest posterior density (HPD) interval TMRCA values calculated in BEAST are shown in each tree nodes. Branch lengths are proportional to time, with the horizontal axis given in millions of years. Asterisk: ‘orphan haplotypes’ (only found in foraging aggregations).

The complete dataset including worldwide rookeries and foraging aggregations of hawksbill turtles resulted in 126 control region haplotypes. Bayesian phylogenetic analysis indicated the presence of nine major mtDNA clades with the TMRCA for all lineages estimated at 5.02 mya (95% highest posterior density interval: 7.12-3.63 mya), when the Atlantic and Indo-Pacific lineages diverged at the early Pliocene (Figure 1). The first lineage in the Indo-Pacific basin to split was clade I at approximately 3.77 mya (95% HPD interval: 5.82-1.99 mya) in the middle Pliocene. The other clades (IIA, IIB, III, IV and V) diverged in the late Pliocene or early Pleistocene. The lineage Indo-Pacific II was split into two clades (IIA and IIB) at about 0.41 mya (95% HPD interval: 0.75-0.16 mya), of which one was reported in foraging aggregations of Atlantic Ocean. The two main Atlantic lineages (clades I and II) diverged more recently, about 1.28 mya (95% HPD interval: 2.16-0.56 mya), followed by a more recent split in clade Atlantic II (A and B) dated at 0.57 mya (95% HPD interval: 0.99-0.23 mya).

**Figure 2:**
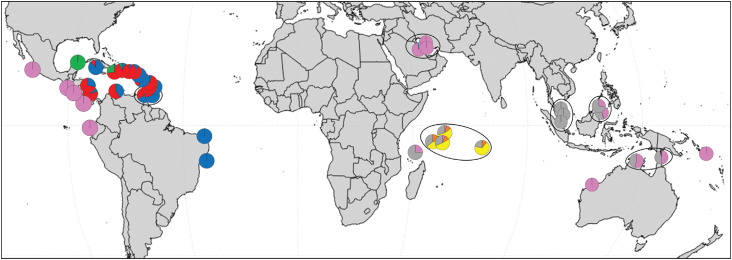
Frequencies of control region haplotypes (739 bp) from each of nine mtDNA lineages in the hawksbill turtle rookeries. Haplotypes were grouped according to the genetic lineage identified by phylogenetic analysis (see Figure 1) and the pie charts show the proportion of individuals from each rookery that belongs to each clade. Major geographical groups are surrounded by an ellipse on the map (see Table S2 for geographical group names).

The genetic diversity of hawksbill turtles was compared between Atlantic and Indo-Pacific populations. The haplotype frequencies per rookeries are shown in Figure 2 and Table S2. The diversity parameters are higher in the Indo-Pacific basin in relation to the Atlantic basin (Table 1). Spatial analysis of molecular variance (SAMOVA) revealed substantial differentiation among Indo-Pacific and Atlantic population groups (FCT = 0.635), with 63.5% of the variation partitioned between groups.

The FST test (based on haplotype frequencies) indicated genetic differentiation among 22 groups of populations, while the exact test showed population differentiation among 25 rookery groups (Table S4). The genetic variance of Indo-Pacific populations was maximally differentiated when they were clustered in five geographical groups, when 65.4% of the variation was found among the following groups: (1) Persian Gulf, Western Australia, Solomon Islands and Eastern Pacific (Mexico, Nicaragua, El Salvador, Ecuador, Costa Rica and Panama), (2) Northern Territory and North Queensland, (3) Seychelles and Chagos Archipelago, (4) Peninsular Malaysia, and (5) East Malaysia (Table S5). However, the grouping of Atlantic populations was not consistent, suggesting different partitions for each K.

Based on exact tests of population differentiation, FST and SAMOVA analyses, we defined the most appropriated regional groups considering both genetic variation and geographical distribution. Considering that different groupings of Atlantic populations were proposed in different tests, we decided to treat each Atlantic population as independent, except for the two Tobago populations. For Indo-Pacific populations, a consistent grouping of 13 different regional groups was observed for all tests (Figure 2 and Table S2).

**Figure 3:**
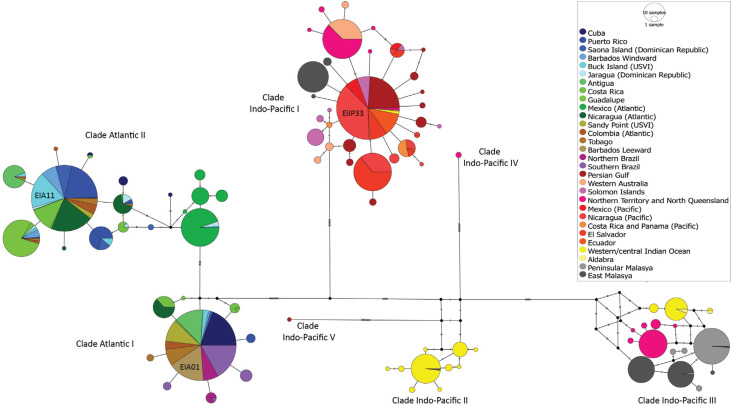
Haplotype (Median Joining) network based on control region of mtDNA (739 bp) found in hawksbill turtle rookeries. Colors correspond to the 30 geographical groups. Small black dots represent median vectors.

The relationships among 739 bp haplotypes of hawksbill rookeries are represented in the mtDNA network (Figure 3). The haplotypes are grouped in seven main clades, which were named according to LeRoux *et al.* (2012) and Vargas *et al.* (2016). Two clades were reported in Atlantic Ocean and five in Indo-Pacific Ocean. Clades Indo-Pacific IV and V are represented each by only one haplotype and they were found exclusively in North Queensland (Australia) and Persian Gulf, respectively. Clades IIA and IIB were found in Western/central Indian Ocean in rookeries of the islands Seychelles and Chagos and one individual was recorded in nesting area of East Malaysia. Haplotype EiIP33 was the most widely distributed haplotype in the Indo-Pacific Ocean, being present in 56% of rookeries. Haplotypes EiA01 and EiA11 were the most common haplotypes in Atlantic (78.9% and 63.1% of rookeries, respectively). The star shaped networks of the clades Atlantic I and II and Indo-Pacific I suggest that the groups have experienced a population expansion. The neutrality tests (Tajima's D and Fu's Fs) support significant changes in population size for hawksbill turtles, indicating an expansion signal for clades Atlantic I and IIA, and Indo-Pacific I, IIA and IIB (p <0.05) (Table 2).

**Table 2:** Neutrality tests in hawksbill turtles from worldwide rookeries and feeding areas, including Tajima's D and Fu's Fs tests based on mtDNA haplotypes (739 bp). N: sample size. Significant P-values are shown in bold.

Clade	N	Tajima's D	P-value	Fs	P-value
Atlantic I	886	-1.6828	**0.007**	-17.08419	**< 0.0001**
Atlantic IIA	673	-1.50708	**0.025**	-10.30654	**0.006**
Atlantic IIB	314	-0.20452	0.483	-1.71103	0.26
Indo-Pacific I	1282	-1.87529	**0.004**	-27.8181	**< 0.0001**
Indo-Pacific IIA	38	-1.53275	**0.046**	-4.76049	**< 0.0001**
Indo-Pacific IIB	65	-1.78534	**0.008**	-6.16794	**< 0.0001**
Indo-Pacific III	294	-0.29422	0.457	-4.01407	0.143
Indo-Pacific IV	7	0.68731	0.809	1.70199	0.753
Indo-Pacific V	1	0	1	–	–

Some haplotypes are exclusive of unique populations or exhibited a very limited distribution. For example, Indian Ocean rookeries of Seychelles and Chagos present 13 exclusive haplotypes, Persian Gulf exhibits 11 exclusive haplotypes, and three of four haplotypes found in nesting areas along the Brazilian coast are also exclusive.

The frequencies of control region 739 bp haplotypes in foraging aggregations are shown in Table S3. Seventeen foraging aggregations were surveyed in Indo-Pacific Ocean, being 12 in the Eastern Pacific coast, four in Malaysian islands and one in North Queensland in Australia. In the Atlantic Ocean, 23 foraging aggregations were analyzed, being six in Southwest, three in Eastern and 14 in Northwest Atlantic (see Table S3).

Eastern Pacific presents all the haplotypes from clade Indo-Pacific II. Southwest Atlantic exhibits a prevalence of haplotypes from clade Atlantic II, while haplotypes of both clades are present in Northwest Atlantic. Foraging aggregations in East and Southwest Atlantic present haplotypes without known origin (EiA49, EiA70, EiA75, EiA82 and EiA87), which belong to the clade Indo-Pacific IIA according to phylogenetic analysis. They were typically found in Seychelles and Chagos rookeries and with one record in Malaysia East rookery. The natal origins of 48.1% of the haplotypes registered in foraging aggregations could not be identified, as they had not been registered in rookeries. These ‘orphan’ haplotypes were found in 76 individuals (4.8% of the individuals analyzed).

## Discussion

### Genetic stocks of hawksbill turtles

Combining all the previously published mtDNA haplotypes, we were able to investigate the phylogeographic patterns of hawksbill turtles in a global context. We demonstrated that the mtDNA diversity among hawksbill turtle rookeries was higher in Indo-Pacific basin relative to Atlantic basin. Six out of the nine phylogenetic clades were found in Indo-Pacific rookeries, which correspond to 69.3% of all compiled haplotypes. It is important to note that only western Atlantic rookeries were analyzed, considering that the available data from eastern Atlantic rookeries comprises short sequences of 384 bp (EATL haplotype) preventing their inclusion in these analyses (Monzón-Argüello *et al.*, 2011).

Some rookeries contain haplotypes from several clades, while other rookeries only contain haplotypes belonging to a single clade. This pattern was observed in Atlantic (LeRoux *et al.*, 2012) and Indo-Pacific basins (Vargas *et al.*, 2016), which reveals a history of early population divergence and subsequent secondary contact for the majority of rookeries.

The SAMOVA analysis revealed that 63.5% of the variation is partitioned between Indo-Pacific and Atlantic groups. A similar differentiation was demonstrated by other studies, but only using limited haplotype representation. No haplotype is shared between Atlantic and Indo-Pacific rookeries, indicating that the American and African continents are important barriers directing the current genetic diversity and distribution of hawksbill reproductive populations.

However, mtDNA haplotypes were shared between distant intraoceanic rookeries. No significant genetic differentiation was observed in FST and exact tests between Colombia and United States Virgin Islands, located about 1000 km apart in the Atlantic Ocean. In the Indo-Pacific basin, widely distributed rookeries throughout the Persian Gulf and Eastern Pacific were clustered together in SAMOVA analysis even when a number of population groups of K=15 was simulated (Table S5).

Previous studies demonstrated a positive but weak correlation between genetic and geographic distances of hawksbill rookeries in Atlantic (LeRoux *et al.*, 2012) and Indo-Pacific basins (Vargas *et al.*, 2016). They suggested that philopatric behavior is important to determine genetic stocks, but it occurs at variable scales and depends on the geographic location and the influence of oceanic currents. This finding makes the phylogeographic patterns more complex, refusing the isolation by distance model that is predominant in the literature.

The connectivity among geographically distant rookeries from Northwest and Southwest Atlantic suggests wide dispersal events. Brazilian populations present four haplotypes, being three exclusive haplotypes derived from EiA01 haplotype. Indeed, Vilaça *et al.* (2013) suggested that EiA01 should be the ancestral haplotype for the Brazilian rookeries.

Populations of the Indian Ocean were clustered in two groups: Persian Gulf and Seychelles-Chagos, which present strong genetic differentiation with high numbers of exclusive haplotypes (11 and 13, respectively). Natoli *et al.* (2017) suggest that the Persian Gulf population originated from a single founder event, followed by population expansion.

Only over the last years, hawksbill rookeries from the Eastern Pacific were examined (Gaos *et al.*, 2016, 2018; Zuniga-Marroquin and Monteros, 2017). They exhibited low genetic diversity and 75% of the haplotypes are exclusive to the region. Haplotypes EiIP106 and EiIP108 were described as unique to the rookeries in mangrove estuaries, a particular hawksbill's behavior found in Pacific Central America (Gaos *et al.*, 2017). Nevertheless, Natoli *et al.* (2017) reported haplotype EiUAE08 in the United Arab Emirates, which matches with the haplotype EiIP106 based on the 739 bp sequence. The duplicity in the haplotype names precluded the detection of a possible origin of the Eastern Pacific haplotype, conducting to uncertain conclusions of species dynamics. The identification of exclusive haplotypes is important to identify the origin of individuals captured in foraging aggregations, since an adequate compilation of all the haplotype diversity is fundamental to suggest endemic origin.

The star shaped network of the clades and the neutrality tests suggest that the groups have experienced a population expansion (Figure 3). Signals of demographic expansion have been previously found for turtles from the Persian Gulf (Vargas *et al.*, 2016; Natoli *et al.*, 2017), Caribbean (LeRoux *et al.*, 2012), Mexican Pacific (Zuniga-Marroquin and Monteros, 2017) and Brazil (Vilaça *et al.*, 2013). In the Persian Gulf and Eastern Pacific, this signal is attributed to a recent colonization event.

In a smaller geographical scale, significant genetic differentiation between nearby hawksbill's rookeries is also commonly observed. Two separate demographic units were recognized for Northern (Ceará and Rio Grande do Norte) and Southern (Bahia and Sergipe) Brazilian nesting areas (Vilaça *et al.*, 2013). In the Persian Gulf, a population boundary was found between Northern (Iran NW) and Southern regions (United Arab Emirates, Iran SE and Saudi Arabia) (Natoli *et al.*, 2017). In the Caribbean, hawksbill's rookeries on the leeward and windward side of Barbados, separated by 30 km, are genetically distinct (Browne *et al.*, 2010). Whilst Buck Island is not differentiated from Barbados rookery (750 km away), it is demographically distinct from Sandy Point in USVI, located 40 km away (Hill *et al.*, 2018). In short, it is not possible to establish distinct demographic units based simply on geographic distance.

Several particularities deserve additional attention in the understanding of the population structure and dynamics of hawksbill turtles. Foraging aggregations in Puerto Rico have revealed temporal variation in haplotype frequencies (Velez-Zuazo *et al.*, 2008). Australian rookeries located 800 km apart exhibited different nesting timing (Vargas *et al.*, 2016). Nesting sites located in a mangrove estuary were recently reported in Eastern Pacific, which represent a new reproductive habitat for hawksbill turtles (Gaos *et al.*, 2017). These peculiar characteristics highlight the complexity of species behavior that should be considered in the delimitation of effective management units and conservation programs.

### Migration patterns

The lack of knowledge on migratory patterns and the origins of juvenile foraging aggregations due to the difficulties in tracking individuals is a serious conservation issue for hawksbill turtles. Using a global dataset based on previously published mtDNA haplotypes, we were able to identify new connections between populations and knowledge gaps that should be prioritized in order to clarify the species dynamics.

**Figure 4:**
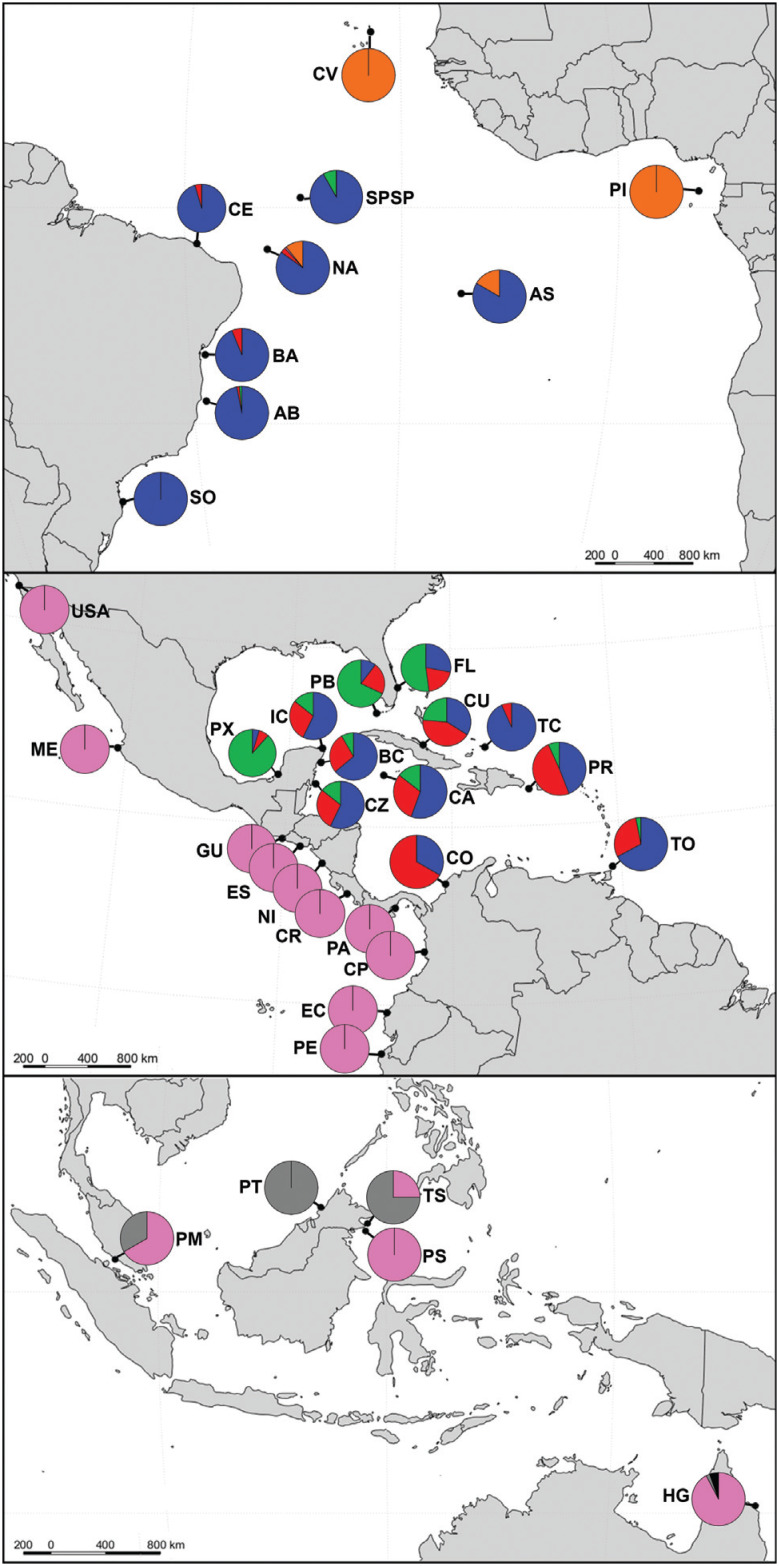
Frequencies of control region haplotypes (739 bp) in the foraging aggregations of Caribbean and Eastern Pacific (upper), South and Eastern Atlantic (middle) and Indo-Pacific basins (lower). The haplotypes were grouped and colored according to the genetic lineage identified by phylogenetic analysis (see Figure 1). See Table S3 for location abbreviations.

The foraging aggregations of Northwest and Southwest Atlantic are well characterized. Brazilian and Caribbean regions share haplotypes, but there is a prevalence of haplotypes from clade Atlantic II at the Brazilian coast, while haplotypes of both clades Atlantic I and II are present in the Caribbean. Foraging aggregations in the Southwest Atlantic present five haplotypes that belong to an Indo-Pacific lineage according to the phylogenetic analysis (Figures 1 and 4). Three of these haplotypes (EiA49, EiA70 and EiA75) are ‘orphans’, while haplotype EiIP16 was reported in rookeries of Seychelles and Chagos Islands and EiIP33 is broadly distributed in the Indo-Pacific basin (Vargas *et al.*, 2016). The presence of these Indo-Pacific haplotypes in the Atlantic Ocean foraging grounds is a clear evidence of a transoceanic movement of individuals.

All the 739 bp haplotypes found in foraging aggregations of Cape Verde and Príncipe Island (EiA49, EiA82 and EiA87) were never registered in rookeries. According to the phylogenetic analysis, they belong to clade Indo-Pacific IIA, which is present in rookeries from Seychelles and Chagos Islands and there is also a record of one individual in the nesting area of East Malaysia. Interestingly, considering the mtDNA data of shorter sequences (384 bp), the clade Indo-Pacific IIA is also found in the rookeries of East Atlantic. Monzón-Argüello *et al.* (2011) reported the presence of females nesting in Príncipe Island off the African coast with haplotype EATL (384 bp), which matches with haplotypes EiA70, EiIP76 and EiIP16 of clade Indo-Pacific IIA. The authors suggested that Príncipe Island was probably colonized by migrants from the Indian Ocean via the Cape of Good Hope in southern Africa. Bowen *et al.* (2007) also proposed that a rare dispersal event from the Indian Ocean into the Atlantic Ocean should have occurred in late Pleistocene. Furthermore, high genetic distinctiveness is observed in the rookeries and foraging aggregations in the Eastern Atlantic regarding the Western Atlantic (Monzón-Argüello *et al.*, 2011).

Shamblin *et al.* (2014) reanalyzed some individuals of loggerhead turtles that had shorter haplotypes available, generating longer sequences that yielded better resolution in population structure. The same is desired for some populations of hawksbill turtles, especially those from Eastern Atlantic, in order to include individuals from the Eastern Atlantic in a robust analysis and to investigate their potential phylogenetic connection with Indo-Pacific hawksbill lineages, as well as to allow a better assignment of the origin of individuals from foraging aggregations.

The foraging ground in Ascension Island is composed by sea turtles originated from Western and Eastern Atlantic rookeries. Simulations of physical transport using an ocean circulation model showed that passive dispersal influencing Ascension Island ground is primarily from the East, involving rookeries along Western Africa and, potentially, the Indian Ocean (Putman *et al.*, 2014).

The integration of ocean drift models and genetic surveys have allowed identifying the influence of oceanic currents in the genetic diversity observed in foraging areas. Aggregations along the Brazilian coast that are influenced by the South Equatorial/North Brazil Current present a differentiated and higher genetic diversity regarding aggregations influenced by the southward-flowing Brazil Current (Vilaça *et al.*, 2013; Proietti *et al.*, 2014). The dispersal patterns of Caribbean hawksbill juveniles vary from regionally constrained groups to mixed stocks with broadly distributed individuals, depending on the local and regional current influences (Blumenthal *et al.*, 2009). For example, foraging areas at Caribbean Mexico are compound by a mix of individuals, while areas at Gulf of Mexico are dominated by self-recruited individuals, due to the effect of the Loop Current and its associated gyres (Labastida-Estrada *et al.*, 2018).

In the East Pacific Ocean, all the juvenile individuals sampled in foraging aggregations present haplotypes from clade Indo-Pacific I. Gaos *et al.* (2017) found that these individuals use foraging grounds in the region of their natal beaches, a behavior called natal foraging philopatry. In contrast, Vilaça *et al.* (2013) showed that rookeries and foraging aggregations found along the Brazilian coast are distinct demographic units, mostly due to a larger genetic diversity and presence of Indo-Pacific haplotypes found in the foraging grounds. In general, there is a significant correlation between the foraging population composition and proximity of the corresponding rookeries, but this pattern is not absolute and foraging aggregates can frequently connect individuals from distant rookeries (Bowen *et al.*, 2007).

A high proportion of haplotypes (48.1%) have no known source rookery, which may suggest that there are many rookeries not yet studied. Further sampling of “genetically unknown” rookeries is needed to identify haplotype origins and improving mixed stock analysis (MSA) resolution of foraging grounds. For example, Labastida-Estrada *et al.* (2018) recently performed the genetic characterization of Mexican rookeries and revealed the origin of the haplotype EiA24, previously identified as ‘orphan’ by Pérez-Bermúdez *et al.* (2017). This finding changes the knowledge about the most important source of hawksbill contributions to Cuban foraging aggregations.

Satellite telemetry data can provide fine-scale information about breeding, foraging and migration of sea turtles in different life stages (Hart *et al.*, 2019). The tracking of female hawksbills showed relatively short post-nesting migrations towards foraging grounds in the Pacific and Atlantic basins (Cuevas *et al.*, 2008; Parker *et al.*, 2009; Gaos *et al.*, 2012; Marcovaldi *et al.*, 2012), but long-distance migrations were also recorded (van Dam *et al.*, 2007; Hart *et al.*, 2019). Acoustic telemetry monitoring of juvenile hawksbills in Belize showed high site fidelity over months to years with occasional wide range use of the atoll (Chevis *et al.*, 2017). However, tracking data is rare and restricted to a few regions worldwide (Marcovaldi *et al.*, 2012). There are several limitations to the remote tracking, as the high cost and brief periods coverage, as well as reduced sample size resulting in poor population-level inference.

Flipper tags and recapture data can also contribute to track hawksbill migratory patterns. For example, Eastward transatlantic movements of juveniles were confirmed when a juvenile hawksbill tagged in a feeding ground at Atol das Rocas in Brazil was captured in Senegal (Marcovaldi and Filippini, 1991).

Hawksbill turtles feed mostly on sponges and forage at or close to coral reef habitats. The migration patterns and the formation of foraging aggregations is likely related to coral reef distribution, as areas with food availability allow turtles to remain resident for long periods (Marcovaldi *et al.*, 2012). However, anthropogenic threats coming from pollution, overfishing and disease have caused a relatively recent coral reef collapse (Pandolfi *et al.*, 2003), which directly impact hawksbill turtles. Thus, conservation strategies aiming to conserve both hawksbills and coral reefs should be broadly considered (Troëng *et al.*, 2005).

Our review and analyses suggest historical long-distance migration and transoceanic connectivity between hawksbill populations. This reinforces the need for extensive and continuous sampling to improve our understanding of the connections between rookeries and foraging aggregations in order to promote adequate protection for hawksbill turtles.

### Phylogenetic history and divergence time

The hawksbill turtle exhibits a deep divergence between Atlantic and Indo-Pacific lineages dating to the early Pliocene, which may reflect the presence of two gene flow barriers: (1) the Isthmus of Panama and (2) the South Africa, where the cold-water Benguela Current disrupts the flow between the Indian and Atlantic oceans (Bowen and Karl, 2007).

The divergence of Indo-Pacific lineages of hawksbill turtles occurred during or subsequent to the early Pliocene. The clade Indo-Pacific I, currently the most widely distributed lineage, is the early diverging lineage in the Indo-Pacific basin, separated from the remaining clades at approximately 3.77 mya. Vargas *et al.* (2016) hypothesized that this could be associated with extreme changes in the Indo-Pacific oceanic currents that happened at this epoch, as a result of continental movements (Australia and New Guinea), the formation of the Isthmus of Panama and the cooling after the warmer Miocene (Cane and Molnar, 2001; Hall 2002).

In late Pliocene or early Pleistocene, the split of other Indo-Pacific lineages (II, III, IV and V), as well as the Atlantic clades (I and II), occurred in parallel with the glacial cycles. The climatic changes during this period affected the sea level and temperature, as well as the oceanic currents, which are expected to impact the gene flow patterns of hawksbill turtles and other marine species (Reece *et al.*, 2005). For example, during Pleistocene glaciations, populations were likely isolated in warmer tropical refuges, accumulating genetic differences (Encalada *et al.*, 1996; Gaos *et al.*, 2016). The colonization of new areas and subsequent demographic expansion were possible during the warmer interglacial periods due to the increase of the sea level and temperature. Thus, population contractions (during glaciation) and expansions (during interglacial times) throughout the Pleistocene have been suggested to drive the complex demographic history of hawksbill turtles (LeRoux *et al.*, 2012).

Atlantic Ocean lineages diverged more recently than any Indo-Pacific Ocean lineage. Given the greater extension of the Indo-Pacific Ocean in warmer and stable temperatures, this basin probably presented more potential refuges during the Pleistocene compared to the Atlantic Ocean (Carpenter and Springer, 2005; Hoareau *et al.*, 2012). Conversely, Atlantic lineages could remain longer connected in the equatorial region, having diverged later.

We found that the lineage Indo-Pacific II is compound by two clades (IIA and IIB). Based on short mtDNA sequence data, the clade Indo-Pacific IIA is present in rookeries of Príncipe Island in Atlantic Ocean (Monzón-Argüello *et al.*, 2011). It is an evidence of a secondary dispersal from the Indian Ocean into the eastern Atlantic Ocean during the late Pleistocene, which should still be confirmed with larger mtDNA sequences.

As depicted in Figure 1 and confirmed by Tajima's D and Fu's FS tests, almost all Atlantic and Indo-Pacific lineages experienced, nearly concomitantly, rapid expansions since the middle-late Pleistocene, during the last 1 million years. Climate change linked to sea level and temperature fluctuations (together with possible changes of the currents) were likely responsible for processes of vicariance, demographic expansion and secondary contact of populations, leading also to the recolonization of new areas during warmer periods (Reece *et al.*, 2005). In our results, the multiple mtDNA radiations for many divergent lineages shown in the phylogenetic tree towards the late Pleistocene suggest that hawksbill turtles have experienced rapid population expansions after severe bottlenecks, which is compatible with temperature cycles of this period.

## Conclusions

The global phylogeographic pattern of hawksbill turtles can be interpreted as two highly divergent major lineages split between Atlantic and Indo-Pacific oceans during the early Pliocene. Following, both major oceanic lineages experienced population contractions and expansions associated with the climatic oscillations and oceanic current changes during the Pliocene or early Pleistocene, which resulted in the separation of different mtDNA lineages with some intraoceanic geographic structure. Migrations and long-distance dispersal events were likely responsible for the partial homogenization of some distant rookeries. During the late Pleistocene, intraoceanic population expansions and a likely transoceanic dispersal from Indian to Atlantic Ocean were observed, establishing a recent separation between East Atlantic/Indo-Pacific and West Atlantic Ocean basins.

This work demonstrated the importance of detailed analyses including the global distribution and diversity of mtDNA lineages of hawksbill turtles. Global demographic patterns were being missed out due to the use of non-representative data or duplicate haplotype names. The systematic exploration of published data allowed achieving relevant conclusions about hawksbill's demography, phylogeography and phylogeny. This shows that worldwide data compilation and standardization can be highly relevant for future studies and for species conservation. Furthermore, this work highlights the need to consolidate international efforts and cooperation to investigate the life history and demographic patterns of hawksbill turtles in a global perspective.

## Supplementary material

The following online material is available for this article:

Table S1 The variable positions for mtDNA control region haplotypes to hawksbill turtle, the accession numbers of Genbank Database, the corresponding haplotypes based on shorter sequences (384 or 480 bp) (Bass *et al.*, 1996; Diaz-Fernández *et al.*, 1999) and the ambiguous haplotype names.

Table S2 Counts of mtDNA control region haplotypes (739 bp) per Hawksbill turtle rookeries. The localities are presented according to the Ocean basin and geographic groups. The data was compiled from the literature and the references are provided per locality.

Table S3 Counts of mtDNA control region haplotypes (739 bp) per Hawksbill turtle foraging aggregations. The localities are presented according to the Ocean basin and geographic groups. Underlined haplotypes are only found in foraging aggregations (‘orphan’ haplotypes). Cells highlighted in grey correspond to individuals reported in Atlantic Ocean that present haplotypes belonging to Indo-Pacific lineage. The data was compiled from the literature and the references are provided per locality.

Table S4 Population pairwise FST based on haplotype frequencies (above diagonal) and P-values of exact tests of population differentiation method (below diagonal) between hawksbill turtle rookeries. (*) Indicate nonsignificant FST values (p>0.05). (+) Indicate significant P-value (p<0.05). (-) Indicate non-significant P-value (p>0.05).

Table S5 F-statistics (FSC, FCT, and FST) associated to the K groups calculated using mtDNA control region of hawksbill turtles from Indo-Pacific rookeries.
