# Supplementary material for: Global phylogeography of the critically endangered hawksbill turtle (Eretmochelys imbricata)
Source: Genet Mol Biol. 2020 Jun 10;43(2):e20190264. doi: 10.1590/1678-4685-GMB-2019-0264 (PMC7288670; doi:10.1590/1678-4685-GMB-2019-0264)

Genetics and Molecular Biology 43, 2, e20190264 (2020)

Supplementary Material to "Global phylogeography of the critically endangered hawksbill turtle (*Eretmochelys imbricata*)"

Table S5 - F-statistics (Fsc, Fct, and Fst) associated to the K groups calculated using control region of mtDNA of hawksbill turtles from Indo-Pacific rookeries.

K	Fct	Fst	Fsc
**2**	0.55776	0.70804	0.33982
**3**	0.60056	0.69164	0.22802
**4**	0.65457	0.68440	0.08635
**5***	0.65472	0.67233	0.05099
**6**	0.65432	0.67041	0.04653
**7**	0.65342	0.65860	0.01496
**8**	0.65229	0.65746	0.01486
**9**	0.65063	0.65661	0.01710
**10**	0.64879	0.65584	0.02009
**11**	0.64636	0.65199	0.01592
**12**	0.64285	0.65113	0.02318
**13**	0.64000	0.64840	0.02335
**14**	0.63643	0.63900	0.00707
**15**	0.63365	0.63840	0.01297

*Geographical groupings based on control region of mtDNA to hawksbill turtle populations from Indo-Pacific Ocean identified by the SAMOVA with K=5.

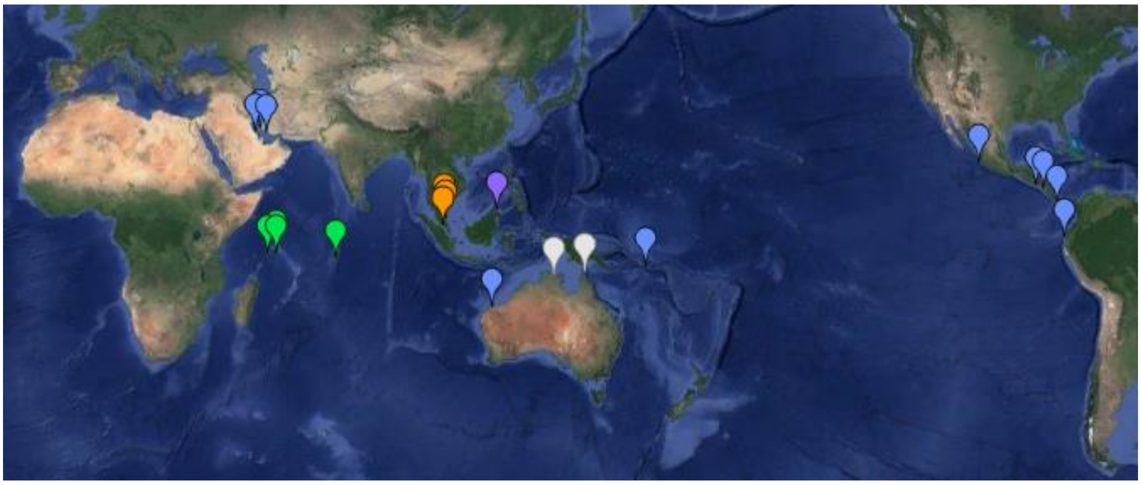

Supplement: Supplementary file 4 [file 1415-4757-GMB-43-2-e20190264-suppl5.pdf]
